# Biotic activity of Ca^2+^-modulating non-traditional antimicrobial and -viral agents

**DOI:** 10.3389/fmicb.2013.00381

**Published:** 2013-12-12

**Authors:** Kevin B. Clark

**Affiliations:** Research and Development Service, Veterans Affairs Greater Los Angeles Healthcare SystemLos Angeles, CA, USA

**Keywords:** antiifective, bacteria, calcium channel blockers, drug design and development, pathogen toxins, probiotic, protozoa, viruses

## Introduction

Combined serendipitous and rational drug-design and -retasking approaches continue to identify many natural and synthetic substances with multipurpose therapeutic properties (Clark, 2013a). Among these substances are Ca^2+^ modulators capable of attenuating the transmission and severity of viral, bacterial, fungal, and protozoal infections (Clark and Eisenstein, 2013; Clark et al., 2013). The majority of purported Ca^2+^-modulating antiinfective compounds belong to the functional drug class termed Ca^2+^-channel blockers, including traditional synthetic 1,4-dihydropyridines, phenylalkylamines, and benzodiazepines long approved and marketed for various human and animal cardiovascular and neurological indications (Clark and Eisenstein, 2013; Clark et al., 2013). Additional Ca^2+^-modulating (putative) antiinfective substances, such as artemisinin, caloxin, dantrolene, cyclosporin A, and FK506, can be further categorized within a broader set of natural and synthetic compounds that affect operation of Ca^2+^ channels, transporters, exchangers, and/or protein sensors of both hosts and infectious agents (Clark and Eisenstein, 2013; Clark et al., 2013). Notably, depending on chemical structure, site, and mechanism of chemical action, and delivered chemical concentrations, these and other non-traditional antimicrobial and -viral compounds, many of which are expressed by pathogens themselves, may instead exert helpful trophic effects on hosts, their symbiotic microbiota, and harbored mutualistic copathogens. The reasons for such biphasic drug-response profiles partly derive from how pathogens evolved to parasitize host Ca^2+^-dependent functions and resources, yielding insights into devising better antiinfective treatment regimens and new valued probiotic medicines.

## Pathogen usurpation of host Ca^2+^ systems

Viruses, bacteria, fungi, and protozoa evolved the strong obligate parasitic strategy of hijacking host systems to augment their comparatively primitive genomic, epigenomic, and somatic capabilities, thereby facilitating infectious disease adaptation and propagation. Though infectious agents coopt many different host systems, few are more significant than host intracellular Ca^2+^ signaling pathways. Free intracellular Ca^2+^ serves as an intermediate between sensory input and response output for all known cellular life. Its ubiquitous presence within cells of diverse phylogeny and function makes Ca^2+^ an essential messenger for controlling host-cell stress responses, fate and death, synaptic plasticity, homeostasis, motility, bioenergetics, growth, morphogenesis, immunodefenses, protein modification and transport, cytoskeletal polymerization, endosome formation, and various other host processes (Clark and Eisenstein, 2013; Clark et al., 2013). Therefore, the ability of microbes to preferentially control host intracellular Ca^2+^ pathways enables them to optimize the timing and effectiveness of infection stages against barriers to invasion, pathogenesis, proliferation, and release (Moreno and Docampo, 2003; TranVan et al., 2004; Kozubowski et al., 2009; Zhou et al., 2009; Clark and Eisenstein, 2013; Clark et al., 2013).

Pathogens, mainly via toxic proteins and lipopolysaccharides, manipulate host intracellular Ca^2+^ systems by modulating (1) ligand- [e.g., N-methyl-D-aspartate receptors (NMDAr)] and voltage-gated (e.g., L-, N-, P/Q-, R-, and T-type receptors and Bsc1, Cch1, and NaChBac receptors) channels that permit Ca^2+^ entry from extracellular spaces, (2) upstream first or second messengers (e.g., inositol 1,4,5-trisphosphate (IP_3_), AMP-activated protein kinase, and mitogen-activated protein kinase pathways), (3) ion- (e.g., Ca^2+^/H^+^ and Na^+^/Ca^2+^ exchangers) and ATP-dependent (e.g., sarcoplasmic-endoplasmic-reticulum (SERCA) and plasma-membrane (PMCA) ATPases) Ca^2+^ pumps that sequester or extrude free cytosolic Ca^2+^, (4) ligand-gated channels (e.g., IP_3_ and ryanodine receptors) and peptidergic porins (e.g., amoebaporins, aquaporins, and PorB) responsible for store-operated Ca^2+^ mobilization and leakage, and (5) downstream host Ca^2+^ binding proteins and sensors (e.g., calmodulin, calrectulin, calcineurin, calnexin, and annexin) (Clark and Eisenstein, 2013; Clark et al., 2013). The wide range of host intracellular Ca^2+^ systems influenced by pathogen factors gives microbes remarkable control over the behavior and well-being of humans and animals, including, but not limited to, mental function and psychological state, voluntary and involuntary motor performance, and gastrointestinal absorption and metabolism. Yet, for microbes, the advantages of pathogen-mediated regulation of host intracellular Ca^2+^ systems extend beyond the impact on host health. In the case of viruses, increased host free cytosolic Ca^2+^ levels may promote viral adsorption, structural stability, capsid uncoating, enzymatic activity, replication, assembly, transport, and fusion (cf. Zhou et al., 2009; Clark and Eisenstein, 2013). Whereas, in cases of bacteria, fungi, and protozoa, alterations of host intracellular Ca^2+^ homeostasis is critical for pathogen sensory transduction, cell energetics, infection sequences, stress adaptation, gene expression, toxin biosynthesis and secretion, molecular biomimicry, conjugation and true sexual reproduction, cell motility and tropisms, growth, biofilm formation and cell aggregation, antigenic variation, and morphogenesis and lifecycle transitions (cf. Cyert, 2003; Moreno and Docampo, 2003; TranVan et al., 2004; Kozubowski et al., 2009; Clark et al., 2013).

## Pathogen selective manipulation of host Ca^2+^ systems

To coordinate pathogen needs with operation of host cells, infectious agents must precisely change their host environment to maximize survival, proliferation, and spread with a repertoire of social-like (e.g., cell-cell communication, biofilm formation, cooperative, and competitive coinfection, etc.) and non-social (e.g., phenotypic variation, biomimicry, etc.) phenomena sometimes interpreted as pathogen intelligence (cf. Crespi, 2001; Casadesus and D'Ari, 2002; Ben-Jacob et al., 2004; Hellingwerf, 2005; Marijuán et al., 2010; Clark, 2013b). In regard to host intracellular Ca^2+^ homeostasis, pathogens rely on certain toxins that may either increase or decrease intracellular Ca^2+^ levels depending on stages of infection and host status. Such fine-tuned aptitude for altering host Ca^2+^ systems confers both advantages and disadvantages on hosts in relation to proper cell function and fate. Although most pathogens have evolved suites of toxins to manipulate host processes, including Ca^2+^-mediated ones, the selective fitness of surprisingly numerous single toxin molecules achieves multiplexed pathogen attacks on their host niche. This kind of pathogen intelligence conserves viral, bacterial, fungal, and protozoal resources for highly efficient and integrated host invasion and exploitation.

For example, overexpression of the multifunctional Hepatitus B Virus (HBV) protein HBx activates caspase-dependent cleavage of host Ca^2+^ PMCA, elevating free intracellular Ca^2+^ concentrations (Chami et al., 2003) as well as IP_3_ production and mitochondrial Ca^2+^ uptake during virus replication (Gearhart and Bouchard, 2010a,b; Yang and Bouchard, 2012). Unless competitively antagonized by IP_3_-receptor-inhibitors dantrolene and FK506 or other drug types, temporary stimulation of the endoplasmic reticulum/mitochondrial interface by IP_3_ boosts ATP synthesis and transport for energy-dependent cell processes required during early viral infection stages. However, when mitochondrial Ca^2+^ uptake subsequently exceeds buffering capacity, HBx advances mitochondrial swelling and fragmentation (Chami et al., 2003), making host cells more vulnerable to free radical generation, metabolic stress, and apoptosis prior to viral release. While sequalae are treatable with non-traditional compounds, including dual-active Beta Cell Lymphoma (Bcl)-related proteins (Clark and Eisenstein, 2013), HBV obviously evolved to carefully manage host-cell operation through well-timed, titrated levels of a single toxin, with lower concentrations of HBx causing long-term/short-term positive outcomes for virus/host and higher concentrations of HBx largely causing positive/negative outcomes for virus/host. This sort of versatility for single viral toxins to exploit host Ca^2+^ systems is observed for other viruses, including Human Immunodeficiency Virus type 1 (HIV-1). HIV-1, via the transcription factor Tat, for instance, potentiates Ca^2+^ influx through dihydropyridine-sensitive voltage-gated L-type Ca^2+^ (Lannuzel et al., 1995) and NMDAr channels (Prendergast et al., 2002; Self et al., 2004), leading to host-cell cytotoxicity. By means of the same Ca^2+^ channels, Tat also evokes production of the tumor necrosis factor (TNF)-alpha cytokine, an important compound for HIV-1 replication and pathogennesis (Contreras et al., 2005). Each harmful effect on host cells may be mitigated by voltage-gated L-type Ca^2+^ (e.g., nifedipine) and NMDAr channel antagonists (e.g., memantine). In contrast, Tat, similar to verapamil, inhibits cytotoxic release of serine esterases by blocking the phenylalkylamine-binding site of voltage-gated Ca^2+^ channels (Zocchi et al., 1998). As with protein HBx of HBV, Tat therefore affords HIV-1 with the ability to either facilitate or guard against host-cell death depending on infection stage and location (e.g., molecule-binding site, cell type, and organ). Moreover, besides direct influence over host condition, both HBx and Tat may act synergistically on HBV and HIV-1 infections (Li et al., 2012) as well as provide opportunistic copathogens, such as mycobacteria (Pathak et al., 2010; Toossi et al., 2012), herpesviruses (Huang et al., 2001; Guo et al., 2004; Caselli et al., 2005), and commensal host fungi (Cassone and Cauda, 2012) and coliform bacteria (cf. Diniello et al., 1998; Mani et al., 2007), an (probiotic) enriched or (antiinfective) hostile host habitat affecting communicable disease progression.

Only two among many instances of viral proteins were discussed above to illustrate the powerful biphasic regulation of pathogen toxins in modifying host and infectious agent physiology (cf. Clark and Eisenstein, 2013). A large number of pathogen-associated Ca^2+^-modulating factors exist for bacteria, fungi, and protozoa as well (cf. Clark et al., 2013). These endo- and exotoxins, of which just a few exemplars will be described here for protists, often allow microbes to evade host defenses by usurping membrane repair systems, down-regulating redox immunological responses, mimicking proinflammatory chemokine and cytokine mobilization, and initiating ireversible host programmed cell death. In addition to purely selfish pathogen infective, survival, and reproductive strategies, such compounds may render trophic support and protective immunity for hosts and their microbiota. Prime examples, similar to those also reported for obligate parasitic *Chlamydia*, *Rickettsia*, and *Toxoplasma* species (cf. Romano et al., 2013), come from intracellular protozoan trypanosomes, etiogenic agents of Chagas' disease, sleeping sickness, and other human and animal illnesses. Several substances, a serine endopeptidase, also called a proteolytically generated trypomastigote factor, Tc-Tox, an acidic pore-forming protein, and acidic sphingomyelinase, synthesized and secreted by *Trypanosoma cruzi* induce host plasma-membrane damage, extracellular Ca^2+^ entry, IP_3_ formation, transient store-operated cytosolic Ca^2+^ liberation, and/or cytoskeletal reorganization to assist in parasite internalization and trafficking (Tardieux et al., 1994; Burleigh and Andrews, 1995; Rodríguez et al., 1995; Burleigh et al., 1997; Fernandes et al., 2011). These compounds are only produced during the infective stage of trypanosome lifecycles, when Ca^2+^-dependent, energy-expensive lysosome and endosome recruitment works to restore integrity of pathogen-injured host plasma membranes. To a limited extent, toxin activation of store-operated Ca^2+^ release can be decreased by IP_3_-receptor blockers. But by directly commandeering host membrane-repair systems and subverting intracellular innate immune-surveillance and potent inflammatory signaling pathways, trypomastigotes ensure successful host invasion and maintenance of host structural and biotic reliability for persistent cryptic and latent trypanosome and copathogen disease states, such as those involving multiple trypanosome strains, symbiotic enterobacteria and other Gram-negative bacteria, and entomopathic double-stranded DNA viruses (Peacock et al., 2007; Alam et al., 2012; Lowry et al., 2013). In turn, these processes, directed by identical toxin concentrations used for trypanosome benefit, can present formidable obstacles to other infectious agents, including convergent trypanosome strains (Ulrich and Schmid-Hempel, 2012) and possible *Encephalitozoon* (cf. Leitch et al., 2001) and *Toxoplasma* parasites (cf. Meirelles and De Souza, 1983), which compete for limited shared host resources and/or must overcome toxin-modified host immunoresponses.

## Prospective Ca^2+^-modulating probiotic and other treatment strategies

Repurposed medications which target pathogen capacities to alter host Ca^2+^ homeostasis and vital cell functions, such as traditional Ca^2+^-channel blockers, SERCA-inhibitor artemisinins, PMCA-inhibitor caloxins, and the IP_3_-receptor-inhibitors dantrolene, FK506, and Bcl antiapoptotic compounds (Clark and Eisenstein, 2013; Clark et al., 2013), show efficacious antiinfective effects against both treatable and previous drug-resistant pathogens. Given examples of HBV, HIV-1, and trypanosome infections readily demonstrate how these drugs exert their chemotherapeutic properties through disruption of pathogen attack, reinforcement of compromised host immunity, and trophic support for host operation. Perhaps more significantly, toxins encoded by pathogens also show non-traditional antiinfective and probiotic traits, oftentimes in a concentration-dependent manner. Such highly adaptive cooperative and competitive traits evolved so pathogens can invade, inhabit, and abandon host niches. Many of these multipurpose pathogen toxins modulate Ca^2+^ systems of host cells and host microbiota, including aforementioned viral and protozoan toxins, HBx, Tat, and Tc-Tox, and different pathogen virulence factors, such as mycobacterial (macolide) mycolactone and lipoarabinomannan (Rojas et al., 2000; Snyder and Small, 2003; Vergne et al., 2003; Boulkroun et al., 2010), staphylococcal leukotoxins (Jover et al., 2013), coliform heat-stable enterotoxin B (Dreyfus et al., 1993), and saccharomycete and ascomycete gliotoxins (Niide et al., 2006), to name a few. In some cases, predictable antiinfective properties of pathogen toxins result from mechanisms known for antibiotic drugs, including the streptomycin-analogous (Diniello et al., 1998) polyamine-starving characteristics of Tat (Mani et al., 2007), or from entirely novel mechanisms. Regardless, pathogen toxins with combined antiinfective and biotic qualities provide exciting substrate to begin developing new medicines of broad therapeutic potential and lifespan.
